# Food: Histogenetic and Non-Histogenetic

**Published:** 1858-12

**Authors:** A. G. Thomas

**Affiliations:** Atlanta, Georgia


### ARTICLE II.

*Food: Histogenetic and Non-Histogenetic.* BY A. G. THOMAS, A.M., M.D., Atlanta, Georgia.

WHAT IS FOOD? Whatever nourishes the animal organism. It is aliment, or the integral elements, which compose the structure or support the vital processes of the organs. Food is classified by Liebig as azotized or tissue-making, and non-azotized or respiratory. The azotized being the only nutritive, and the non-azotized, not entering into organized tissues, the non-nutritive substances: food, therefore, being *Histogenetic* or *non-Histogenetic.* This theory of Liebig is adopted by such men as Boussingault, Tiedeman, Draper and Carpenter. That Carpenter, has fully committed himself to this theory, appears from the following, from his "Human Physiology:" "But besides the *histogenetic* materials and oxygen on the one hand, and the various products of the disintegration of the tissues on the other, the blood contains those *non-azotized* substances which are received into it for the purpose of supplying the *pabulum* of the combustive process." I shall now consider whether this popular theory be strictly physiologically true. That food is either nitrogenized or non-nitrogenized is chemically true; and it is also true that man requires aliment both histogenetic and combustible; but that *nitrogen* is *the* only integral histogenetic element, and the tissue-forming value of any substance *depends* upon the presence of nitrogen, I do not think is true for the following reasons:

1st. Whatever element is a component part of a tissue is to that extent tissue-making. Among the various classes of tissues there is one called *adipose tissue*, a name authorized by medical Lexicons and adopted by medical teachers. The very name of this tissue indicates clearly that it is composed largely, if not entirely, of adipose matter, which analysis proves is non-azotized. If, then, non-azotized matter be the principal element of a distinct tissue, how can the medical man affirm that none but azotized matter can be tissue-making?

2d. There are races of men living in climates where the supply of combustible matter needed to sustain the proper temperature of animal heat is very small. The Hindoo lives upon rice and rancid butter. His rice contains only 3.60, in 100 parts, of *gluten* a nitrogenous body, and his butter contains no nitrogen at all. Therefore, non-azotized food *must* furnish the greater part of his nutrition.

3d. No molecule, not a blastederma can exist, unless it contains an appreciable quantity of salts and fatty matter. I cheerfully admit the proposition of Liebig, "that albumen is a necessary element in the organized tissues;" but, it is equally certain that salts, fats and oils, non-azotized substances, are as essential as is albumen. Anatomical investigation proves that no tissue exists, composed wholly of albumen or azotized matter. But, on the other hand, with every stroke of the scalpel, the anatomist meets with fat in nerve, in muscle, in cartilage, in bone, in every tissue. Is it not an admitted fact that nerve-tissue without fat is not normal nerve? Muscle is said to contain in 100 parts, only 25.55 parts of solid matter, and of these 4.25 are fatty matter. In the white substance of the brain, of 100 parts 13.9 are fat. In the grey brain substance, fat is to albumen as 4.7 is to 7.5. Bone cannot be formed unless it contain a certain amount of carbonate of lime.

4th. Water, certainly non-nitrogenous, enters largely into the formation of *blood tissue.* I know not that I shall be allowed to call the blood a tissue, and yet our Physiologists teach us "the blood is the living body." In 1000 parts of this *living body* there are, according to an analysis by M. Lecanu, 785.58 of these parts water, and, *par parenthese*, of the remaining 214.42 parts, there are 6.57 parts of fat and 10 parts of salts. Not to insist, however, that blood is strictly a tissue, I pass on to my fifth reason.

5th. Water is a necessary ingredient in the formation of tissue, and judging from the great quantity of water in the system, it is the most nutritive element. That water is an essential element of animal tissue is abundantly shown by the fact, that the immense proportion of two-thirds of the whole organism is water. The cornea of the eye would not be transparent without water. Respiration depends greatly on the amount of moisture in the lungs, and the tissues would possess no elasticity but for the presence of this universal element. To support these views, I quote from Magendie: "Muscular flesh in which gelatine, albumen, and fibrine are combined, according to the laws of organic nature, and where they are associated with other matters, such as fats, and salts suffices in very small quantities for complete and prolonged nutritlon." I introduce one other author to sustain this position, and that one is none other than the great champion of the theory of nitrogen as exclusively tissue-making—Liebig. It was once asked: "Is Saul also among the Prophets?" It may now be asked: Is Liebig also among the advocates of the theory, that non-azotized substances, as well as azotized, are essential to the formation of animal tissues? Liebig is of age, he can speak for himself. In his Chemical Letters, he says: "Many of the physical properties of the organs or tissues depend on the presence of their non-nitrogenous *constituents*, viz: of water and fat. These bodies assist in the changes and processes by which the organized structures *are formed.* Fat has a share in the *formation* of cells, and on water depends the fluidity of the blood, and of all other juices. So, also, the milk-white color of cartilage, the transparency of the cornea, the softness, plasticity, flexibility and elasticity of muscular fibre and of membranes, all depend on a fixed proportion of water in each case. Fat is a never-failing constituent of the substance of the brain and nerves. Hair, horn, claws, teeth and bones always contain a certain amount of water and fat." If language can be explicit, Liebig has here explicitly affirmed that tissues are dependant on "a certain amount" of non-nitrogenous matter. To do him justice, however, I will state that he has tried to be consistent with the azote theory, by saying, after making the above unmistakable remarks, that these parts of "water and fat are only mechanically absorbed as water in a sponge, or enclosed in drops as fat is in cells, and they may be removed by *mechanical pressure*, or by solvents, without in the least affecting the structure of the parts." To which I reply: If water by itself, or fat by itself, has no vital properties, neither has fibrine nor albumen by itself any vital properties. I have already shown that water is an essential element of tissue, and I apprehend if Liebig, *in propria per sona*, had been subjected to "mechanical pressure," he would have acknowledged that the structure was somewhat affected, long before a tithe of that element which is two-thirds of the human body, had been expressed from his individual organism.

6th. Physiological testimony declares that the fibrine and albumen of the tissues undergo chemical changes in the decay and repair of the system; and that there is a waste of tissue, the result of mechanical exertion. It is a wellknown and universally admitted chemical law, that chemical action evolves heat; it is also a philosophical truth that mechanical pressure, such as rubbing tissues on each other, evolves heat. The conclusion legitimately deduced from these premises, is that the albuminous tissues of the body are heat producers; that the heat-making process is not confined to the non-azotized. This conclusion is supported by the facts of nature; for instance: we learn from those who have travelled largely, that there are races of human beings who live for long periods on food which is highly albuminous, containing little or no fat. Nitrogenous substances must furnish for such races sufficient heat to sustain the temperature of their bodies.

7th. In cold countries great quantities of oil, seal-fat and whale blubber are consumed. Dr. Carpenter tells us that "the Esquimaux and Greenlanders are able to *take in* 20 or 30 pounds of blubber at a meal." While I admit that a portion of this matter undergoes combustion in the body, I would ask Dr. Carpenter if the *whole* of this non-nitrogenous substance is consumed to enable them "to pass a large portion of the year in a temperature below that of our coldest winter," what is it that keeps up the supply of muscular tissue? For it is certain that they must have muscle to enable them to lead very active lives, "spending a great portion of their time in the open air." Besides, Dr. Carpenter informs us that when supplied with twenty or thirty pounds of blubber "they are enabled to pass several days without food," when they again eat the same amount of this, their only food, and again go for several days without food. What supplies the solid tissues of their bodies? I unhesitatingly answer that a portion of this same blubber affords the necessary supply of the tissues. He also says in his Elements of Physiology.—Page 111.—"There is good ground to believe, however, that, for the formation of *all the animal tissues*, the presence of fatty matter, as well as an albuminous compound, is essential,"

I think I have thus proved that azotized substances are heat-makers *and* tissue-makers, and that the non-azotized are tissue-makers, therefore serving other purposes besides making animal heat and "assisting in the elaboration and assimilation of albuminous bodies," they are *histogenetic*.

Having shown that azotized and non-azotized substances are essential as *histogenetic* and *non-histogenetic* elements, I will suggest that *food* should be classified, not chemically, azotized and non-azotized, but physiologically, ORGANIC and INORGANIC; for which idea I am indebted to no less a personage than Dr. William B. Carpenter. After spending some time in developing the other classifications, it would seem unwittingly, he acknowledges the superiority of the latter. On page 250 "Elements of Physiology" Am. Ed., he says: "the organic compounds which have been enumerated as supplying *the various wants* of the system would be totally useless without the admixture of certain inorganic substances, which also form constitutent parts of the bodily frame." With this suggestion I leave the subject, hoping the day is not far distant when physiology "shall see for itself and not for another." When physiologists shall teach physiology as it is, showing that whatever elements support both heat and tissue in the wonderfully and fearfully framed human organism, are both HISTOGENETIC and NON-HISTOGENETIC.

